# Onco-Breastomics: An Eco-Evo-Devo Holistic Approach

**DOI:** 10.3390/ijms25031628

**Published:** 2024-01-28

**Authors:** Anca-Narcisa Neagu, Danielle Whitham, Pathea Bruno, Aneeta Arshad, Logan Seymour, Hailey Morrissiey, Angiolina I. Hukovic, Costel C. Darie

**Affiliations:** 1 Laboratory of Animal Histology, Faculty of Biology, “Alexandru Ioan Cuza” University of Iași, Carol I bvd. 20A, 700505 Iasi, Romania; 2 Biochemistry & Proteomics Laboratories, Department of Chemistry and Biomolecular Science, Clarkson University, Potsdam, NY 13699-5810, USA; whithad@clarkson.edu (D.W.); brunop@clarkson.edu (P.B.); anarsha@clarkson.edu (A.A.); seymoule@clarkson.edu (L.S.); morrisha@clarkson.edu (H.M.); aihukovic@gmail.com (A.I.H.)

**Keywords:** breast cancer (BC), onco-breastomics, eco-evo-devo theories, tumorigenesis, progression

## Abstract

Known as a diverse collection of neoplastic diseases, breast cancer (BC) can be hyperbolically characterized as a dynamic pseudo-organ, a living organism able to build a complex, open, hierarchically organized, self-sustainable, and self-renewable tumor system, a population, a species, a local community, a biocenosis, or an evolving dynamical ecosystem (i.e., immune or metabolic ecosystem) that emphasizes both developmental continuity and spatio-temporal change. Moreover, a cancer cell community, also known as an oncobiota, has been described as non-sexually reproducing species, as well as a migratory or invasive species that expresses intelligent behavior, or an endangered or parasite species that fights to survive, to optimize its features inside the host’s ecosystem, or that is able to exploit or to disrupt its host circadian cycle for improving the own proliferation and spreading. BC tumorigenesis has also been compared with the early embryo and placenta development that may suggest new strategies for research and therapy. Furthermore, BC has also been characterized as an environmental disease or as an ecological disorder. Many mechanisms of cancer progression have been explained by principles of ecology, developmental biology, and evolutionary paradigms. Many authors have discussed ecological, developmental, and evolutionary strategies for more successful anti-cancer therapies, or for understanding the ecological, developmental, and evolutionary bases of BC exploitable vulnerabilities. Herein, we used the integrated framework of three well known ecological theories: the Bronfenbrenner’s theory of human development, the Vannote’s River Continuum Concept (RCC), and the Ecological Evolutionary Developmental Biology (Eco-Evo-Devo) theory, to explain and understand several eco-evo-devo-based principles that govern BC progression. Multi-omics fields, taken together as onco-breastomics, offer better opportunities to integrate, analyze, and interpret large amounts of complex heterogeneous data, such as various and big-omics data obtained by multiple investigative modalities, for understanding the eco-evo-devo-based principles that drive BC progression and treatment. These integrative eco-evo-devo theories can help clinicians better diagnose and treat BC, for example, by using non-invasive biomarkers in liquid-biopsies that have emerged from integrated omics-based data that accurately reflect the biomolecular landscape of the primary tumor in order to avoid mutilating preventive surgery, like bilateral mastectomy. From the perspective of preventive, personalized, and participatory medicine, these hypotheses may help patients to think about this disease as a process governed by natural rules, to understand the possible causes of the disease, and to gain control on their own health.

## 1. Introduction

BC is the most commonly occurring cancer in women worldwide [1] and accounts for 30% of all cancer diagnosed in women [2]. Characterized by extensive genotypic and phenotypic intratumor and intertumor spatio-temporal heterogeneity, BC is a diverse collection of neoplastic diseases and represents a great challenge for predictive, personalized, precision, preventive, and participatory (5P) onco-medicine [3,4]. From a biomedical point of view, one can talk about the many faces of BC evolution [5] that master the perfect art of tumor face-changing [6] based on multiple adaptations of the BC molecular landscape [7], signaling pathways [8], metabolism plasticity [9], risk factors [10], or genes regulation [11], which all together initiate and drive tumorigenesis, progression, and the recurrence of this chameleonic disease. BC heterogeneity is due to differences in the genomic, transcriptomic, proteomic, metabolomic, and epiomic biomolecular features of BC landscape [1]. This review aims to be an approach of BC unity in diversity based on multiple research studies integrated as the onco-eco-evo-devo theory sustained by highly developed omics approaches used in the BC field, which may be integrated into a holistic term called onco-breastomics. 

To better understand the molecular and clinical characteristics of BC, multi-omics approaches and bioinformatics are considered novel frameworks that integrate numerous omics data sets [12]. The scientific terms provided with the -*omics* suffix manage large-scale information summed up in different omes [13], which are known as relevant components from a particular biomolecular subset, according to a definition quoted into an expressive and suggestive editorial titled “*I’m an-omics, you’re an-omics*…” [14]. The great development of omics systemic approaches shows that each *omics* domain reflects not only the sum of elements that constitute the corresponding *ome*, but rather their interactions and the direct and indirect influence on each other [15]. Apart from classical omics domains such as genomics, transcriptomics, proteomics and metabolomics, some new ones are emerging. For example, the concept of “omes-epi-omes” interactions is based on epigenomics, epitranscriptomics, epiproteomics, epichaperomics, and epimetabolomics as epiomics fields that are focused on the epimodifications or epimarks, such as DNA/RNA modifications, post-translational modifications of proteins (PTMs) and metabolite changes under an environmental context [16,17]. For example, phosphoproteomics is focused on phosphorylation as a PTM of proteins and contributes to an understanding of how exposure of BC cells to stressful tumor environments affects the activity of signaling networks involved in metabolic and growth factor signaling [18], and drives the plasticity of cell migration programs contributing to metastasis [19]. Moreover, proteins do not function alone and protein–protein interaction (PPI) networks are context-dependent [20]. PPI networks allow cells to respond to chemical and physical stimuli from intracellular and extracellular environments through stressor-induced protein connectivity perturbations [20,21]. Consequently, interactomics and proteomics approaches may investigate the alterations in protein connectivity induced by stressors, without omitting that interactomes have a high heterogeneity among different BC cell lines/BC subtypes and should be characterized in a wider range of cellular contexts [22]. Taken together, onco-breastomics emerges as a modern biomedical field that may integrate, analyze, and interpret large amounts of complex heterogeneous data, such as various and big-omics data obtained by multiple investigative modalities in BC for understanding the onco-eco-evo-devo-based principles that drive BC progression and treatment.

## 2. Why This Review Is Important?

Cancers have been characterized as “microcosms of evolution”, in which, by “microevolutionary processes”, mutant cells reproduce/proliferate, resist and survive, evade, invade, compete, and cooperate to disperse and to colonize distant organs [23]. It is known that biological, ecological, and evolutionary thinking may provide fundamental, new, and helpful insights into oncological research and cancer therapy, especially based on modeling of cancer development and progression, thus complementing the biomolecular and cell-based approaches [24]. From the perspective of preventive, personalized, and participatory medicine, we consider that future oncological education will need to learn how to use and deal with analytical tools, strategies, and advanced technologies to cultivate the patient to think about their disease as a long process governed by rules found in nature or inside all bio-ecological systems (i.e., ecosystem, species, organism, tissue, cell, embryo etc.) at the intersection of traditional and modern holistic approaches. This is necessary for understanding the complexity of possible causes of disease or to find/activate the body’s inner resources (immunity, mental etc.) or social factors able to improve the survival and even to assure the best possible quality of life in this given condition. It is important to understand that patients can gain control on their own health by educating them to understand and accept the complexity of their disease and therapy [25]. Broadly, bio-medical science is able to develop integrated theories that promote the use of the process-based and data-driven models to assure the treatment of the whole person based on the theory-guided data science, which “introduces scientific consistency as an essential component for learning generalizable models” [26]. These integrated eco-evo-devo theories may help practitioners to better diagnose and treat BC. For example, it is important for clinicians to be informed and to use validated and the most appropriate biomarkers assessed in non-invasive liquid biopsies (blood, urine, milk, tears) that reflect the pathological biomolecular or cellular landscape of the primary or metastatic tumors (river continuum concept vs. breast cancer cell/proteomic continuum concept) or that could help for an accurate classification or BC staging, to avoid mutilating preventive surgery, like bilateral mastectomy. Thus, the multi-omics-based investigation of BC, especially the integrated approaches, are useful for the identification of new biomarkers in liquid biopsies to develop non-invasive investigation or to monitor different BC treatments. In this context, the similarities between the metastatic cascade and processes that occur in natural organisms or ecosystems may serve as the basis of “ecological reconstruction or restauration” into the breast or at secondary sites affected by the tumoral process. The patient must know the mechanisms that drive the BC development and the natural- and medical-based tools that can be handled to better survive with, understand, and treat this chameleonic disease.

## 3. Onco-Eco-Evo-Devo Breastomics

### 3.1. BC Is a Genomic Disease

Cancer is a disease of the genome [27]. The development of many tumors starts at the cell level through a process of somatic mutation that causes genetic variation/instability and arises through natural selection which acts as purifying selection that eliminates the deleterious mutations, or as positive selection, which maintains the functionally advantageous mutations, which allow, from a neoplastic perspective, aberrant proliferation, invasion, and metastasis [28,29,30]. Most tumoral clones share genomic rearrangements and cancer driver gene mutations, illustrating that such events may occur early in the cancer evolutionary process [31]. However, Ostrow et al. (2014) showed that the cancer evolutionary process, dominated by positive selection of somatic mutations at a cell level, differs from organism’s evolution, which is dominated by purifying selection affecting germline (hereditary) mutations at organismal level [29]. As a clonal evolutionary process, cancer is also caused by the successive accumulation of epigenetic alterations that lead to tumorigenesis, progression, dissemination, and treatment resistance as well [32]. BC is characterized by a high genomic instability expressed in somatic gene mutations, copy number alterations (CNAs), and chromosome structural rearrangements caused by defects in DNA damage repair (DDR), transcription, DNA replication, telomere maintenance and mitotic chromosome segregation [33,34]. Multi-omics analyses, including genomics, transcriptomics, proteomics, and even single-cell molecular profiling, are essential to characterize the molecular mechanisms of multi-omics regulation in BC [35], or to identify -omics differences in BC-related transcriptional regulatory network gene hubs between certain ethnic groups [36].

Multiple BC genes are known to be associated with fertility, aging, longevity, environmental adaptation, and evolution, their roles extending from cells to individuals and to populations [37]. Highly penetrant breast cancer susceptibility genes 1 and 2 (*BRCA1* and *BRCA2*) are found in a wide variety of organisms [38], also being the most common germline mutated genes, while the phosphatidylinositol-3-kinase (*PIK3CA)* is the second most common somatic mutated gene in BC patients, just after *TP53* [30]. Furthermore, *BRCA1/BRCA2* downregulation and *PIK3CA* overexpression may be targeted for BC therapy [30]. Both *BRCA1* and *BRCA2* are iconic BC predisposition genes involved in repairing damage to chromosomal DNA, cell cycle control, transcriptional regulation, resulting in genome integrity/stability, so that the high penetrance mutations in these genes lead to a loss of tumor suppressor activities and an increased breast cancer risk (BCR) [39]. Pfeffer et al. (2017) and Li et al. (2022) emphasized that the presence of both *BRCA1* and *BRCA2* homologues and orthologues dates back to 1.2–1.6 billion years ago in animal and plants, with *BRCA2* in fungi as well [38,40]. In 2002, Warren et al. performed, for the first time in non-mammalian species, a structural analysis of the chicken *BRCA2* gene to facilitate identification of functional domains and disease causing mutations [41]. These authors identified that the *BRCA2*-gene products in chicken are poorly conserved with mammalian BRCA2 proteins, but certain domains are much more highly conserved, suggesting a functional significance. However, cancer-associated mutations have been found to occur at conserved sites [38]. Lou et al. (2014) showed that both genes evolved under rapid positive selection during simian primate speciation, despite the fact that it is expected that DNA repair proteins would be evolutionarily conserved over time [42]. Li et al. (2022) showed that *BRCA* germline pathogenic variants that increase the BCR arose in recent human history, after the latest out-of-Africa migration, and the expansion of modern human populations could highly increase the variation spectrum [40]. Michalak and Kang (2018) highlighted a unique and likely pathogenic divergence of the *BRCA2* in *Homo neanderthalensis* relative to other primates, including modern humans, raising a question about cancer susceptibility in the archaic species that were replaced by modern humans 40,000 years ago [39]. Until now, Fu et al. (2022) showed that 1800 mutations have been detected in human *BRCA1* [43]. Mutations in the *TP53* tumor suppressor gene seem to be adaptive for animals living in hypoxic and cold environments or being exposed to starvation [37]. As BRCA1/2 proteins, p53 protein, encoded by the *TP53* gene, plays a key role in tumor suppression by certain mechanisms involved in cell metabolism, cell cycle arrest, DNA repair, genome stability, apoptosis, ferroptosis, and angiogenesis [44]. Almost 80% of triple-negative breast cancers (TNBCs) express an inactive, mutant form of p53 protein, resulting in rapid cell growth and metastasis [45]. *TP53* gene is specific for the Holozoa branch, where its ancestral p63/p73-like genes emerged one billion years ago [44]. Sulak et al. (2016) used an evolutionary genomics/transcriptomics-based approach to show that several large animals have an increase in the copy number/retrogenes of *TP53* gene that protect them against cancer [46]. Kou et al. (2023) applied phylogenomics- and paleogenomics-based approaches to identify the evolutionary origin of TP53 germline pathogenic variants (PVs) in modern humans [47]. These authors observed no direct evidence for the cross-species conservation as the origin of PVs, but revealed that *TP53* germline PVs in modern humans were likely originated in recent human history and partially inherited from the extinct Neanderthals and Denisovans [47]. The *TP53* gene has a different mutation prevalence by race, with African-American women having significantly more TP53 mutations than White women [48].Considered as a cancer susceptibility gene that harbors germline alterations in multiple cancers including BC, the checkpoint kinase 2 (*CHEK2*) is a gene that encodes a protein kinase that may be activated by phosphorylation in response to DNA damage, serving for TP53 stabilization, cell cycle arrest, genome maintenance, and apoptosis [49]. Thus, *CHEK2*, as well as other genes included in the human Fanconi anemia/breast cancer-associated (FANC/BRCA) pathway, which is involved in DNA damage-repair responses, shows an apparent concentration of positive selection [50]. Another essential gene for repairing DNA, the partner and localizer of BRCA2 (*PALB2)* is a tumor suppressor gene, also identified as a susceptibility gene for human BC [51]. As *CHEK2*, *PALB2* also harbors germline pathogenic mutations. Chian et al. (2023) have performed a phylogenetic analysis by tracing *PALB2* gene variants in 100 non-human vertebrates and a paleoanthropological analysis by tracing *PALB2* variants in over 5000 ancient humans, concluding that there is no evidence to support the evolutionary conservation as the source for human *PALB2* pathogenic variants (PVs). *PALB2* benign variants were also highly shared with ancient humans, while the PVs mostly arose in recent human history, in the past 10,000 years [52].

Phosphatidylinositol-3-kinase (PI3K), encoded by the *PIK3CA* gene, is known to promote cell transformation, tumor initiation, proliferation, and resistance to apoptosis [53]. In BC, the PI3K over-activation is correlated with decreased phosphatase and tensin homolog deleted on chromosome ten (*PTEN*) expression that leads to activated and increased levels of AKT, thus promoting cell cycle progression [53]. Furthermore, PTEN is a tumor suppressor and a highly penetrant gene involved in hereditary predisposition to BC, which regulates and extends longevity through the inactivation of PI3K/AKT/FoxO pathway and maintenance of genome integrity in worms, flies, and mammals [54].

Last but not least, BC risk (BCR) differs across ancestry [55]. The presence of cancer-causing mutations in ancient human communities, including *TP53* and *BRCA2* variants, and differences in their frequency among ancient populations, as well as in contemporary ones, have been demonstrated [56]. Bhaskaran et al. (2019) have showed that germline variations in *BRCA1/2* are highly ethnic-specific, so that current Caucasian population-based *BRCA* data is not adequate to represent *BRCA* status in non-Caucasian populations [57]. It is also known that there are evidence that African-Americans (AA) have a higher incidence of early-age onset BC and triple-negative breast cancer (TNBC) than European/white-American (EA) women [58,59].

### 3.2. BC Is a Developmental Disorder

Numerous parallelisms exist between development and cancer [60]. In cancer cells, due to high genomic instability, reactivation of embryonic development process/pathways may contribute to tumor expansion and metastasis formation [61]. Thus, many genes/proteins involved in different stages of embryonic development are dysregulated or mutated in BC cells. It has been demonstrated that BRCA1 plays a biological role in protecting the embryo from oxidative stress [62], as well as the p53 tumor suppressor family members that play roles during mammalian embryonic development [63]. Known as “the guardian of the genome”, p53 activity was first detected at the late blastocyst stage [63]. The zinc-finger transcription factors GATA4, which play important roles in BC progression from an early stage, and GATA6, which is overexpressed in BC and promotes BC cell epithelial-mesenchymal transition (EMT) [64] are essential for embryonic development during gastrulation [65]. Cofre et al. (2019) showed that both developmental pathways in gastrulation and cancer require the ability to respond to environmental stimuli [66]. Thus, the Wnt signaling pathway has been reported to be highly conserved in metazoan animals and to play a critical role in controlling embryo and organ development, as well as in BC cells transformation, where its alterations have been correlated with mutations, amplifications, deletions, DNA methylation, and post-translational modification of proteins (PTMs) [67]. Also, estrogen receptors are expressed in all vertebrates and play significant roles for normal vertebrate embryonic development [68], while estrogen receptor signaling pathways have been deeply associated with hormone-dependent BC (estrogen and progesterone receptor positive), which accounts for two-thirds of breast tumors [69]. Human epidermal growth factor receptor 2 (HER2) is also a gene that encodes a growth factor receptor, which activates intracellular mechanisms that lead to embryonic development, or, when overexpressed or mutated, can lead to the development of cancers, including HER2-positive BC [70].

Some hypotheses suggest that the cellular mechanisms used by placental cells during embryo implantation in human pregnancy, such as angiogenesis, signaling pathways, or migration and invasion, are reused by tumor cells to migrate, invade and spread within the host’s distant organs [71,72,73]. Costanzo et al. (2018) showed that the trophoblast, which forms in the outer layer of the blastocysts, mimics several malignant functions, such as the ability to invade the endometrium and to attach to the uterus wall, to build new vessels connecting fetal to maternal circulation, and to suppress maternal immune responses [61]. Moreover, the cytotrophoblast (CTB) cells undergo a partial EMT when differentiating in extravillous cytotrofoblast (EVT), gaining the capacity to migrate and invade [74]. In this context, it is known that downregulation of epithelial cadherin (E-cadherin) is required to initiate the invasive BC cell phenotypes by EMT [75] similar to the downregulation of this biomarker expression during trophoblast differentiation into its invasive subpopulations [76]. Moreover, a genomic-based study showed that other epithelial markers, like occludin, have been downregulated in EVT compared to CTB, while mesenchymal markers, such as vimentin (VIM), fibronectin (FN) as well as matrix metalloproteinases (MMP2 and MMP9) have been upregulated in EVT compared to CTB [77]. Similarly, VIM [78], FN [79], and MMPs [80] are important EMT-signature proteins that promote BC cell migration and invasion.

Manzo (2019) showed that embryonic stem cells (ESCs) and cancer stem cells (CSCs) share similar characteristics, assuring both embryo development as well as cancer process, resulting in new strategies for cancer research and therapies [81]. Thus, Hadjimichael et al. (2015) highlighted a plethora of molecular signatures which are common in embryonic and cancer stem cells that share many common features, such as pluripotency, rapid proliferation, similar metabolic requirements, and inhibition of differentiation: (i) surface molecules, signaling pathways biomarkers and transcription factors; (ii) gene signatures; (iii) signaling pathways, such as Jak/STAT, Wnt/β-catenin signaling, Hedgehog and Notch, TGF-β, fibroblast growth factor signaling; and (iv) epigenetic regulators, like DNA methylation regulators and chromatin modifiers [82].

Consequently, tumor progression has been successfully compared with implantation and embryo development, which suggests that cancer can be considered a problem of developmental biology [83].

### 3.3. BC Is an Ecological/Environmental Disorder

BC has been characterized as an environmental disease/ecological disorder [84,85]. Lifestyle and environmental factors, as well as hereditary/genetic factors, predict BC development [86]. Eco-oncology, focused on gene-external environment interaction studies, may improve the understanding of the BC biology [87]. Hiatt and Beyeler (2022) show that climate change will affect women’s cancers through various mechanisms, including the effects of air pollution, ultraviolet radiation (UV), environmental toxins, or aberrant food supplies [88]. Shih et al. (2020) sustained that even smartphone long-term use significantly increases the BC risk, due to a close distance between breasts and smartphone, and the habit of smartphone use before bedtime and night [89]. Also, night shift work, exposure to light at night, and circadian disruption may increase the BC risk [90]. The oncogenic viruses have a great role in BC etiology and pathophysiology [91]. In this context, many authors summarized or demonstrated the role of environmental chemicals and other exposure types in BC progression, metastasis formation and recurrence to chemotherapy [92,93,94].

The environment is defined as anything that is not genetic and includes chemical, toxicants, social, and built aspects [85]. Recent evidence indicates that the intrinsic risk factors contribute modestly to the lifetime risk of cancer development, while 70–90% of cancers occur under extrinsic environmental factors pressure [95]. Exposomics is able to characterize all environmental exposures from a variety of external and internal sources, as well as the biological response of an organism to exposures [96]. Exposomics investigations are conducted through the application of omics-based technologies that identify biomarkers of exposure and responses to chemical and biological agents or radiation [97]. Recently, Gao (2023) showed that single-cell exposomics is a revolutionary approach that investigates the interactions between cell and environment stressors at cellular and subcellular level, especially based on spatial and high resolution mass spectrometry (MS) analysis [98]. Thus, recent advancements in single-cell technologies have enabled detection of RNA, by single-cell RNA sequencing (scRNA-seq) [99], proteins, by single-cell proteomics by MS, metabolites, by mass spectrometry imaging (MSI) or secondary ion mass spectrometry (SIMS), and xenobiotics, including drugs, by single-probe single-cell MS or capillary electrophoresis-laser-induced fluorescence, in individual cells, by time-of-flight MS (CyToF), contributing to a better understanding of mammary cell diversity [100] or demonstrating that distinct cell populations express xenobiotic metabolizing enzymes, which indicate variability in xenobiotic metabolism not only between organs, but also at a tissue and cell level [101]. Sellami et al. (2020) showed that nutrigenomics and nutriproteomics are recently developed omics fields that are focused on the interaction between nutrients and human genome/proteome in order to decipher the role of dietary factors in carcinogenesis [102]. Additionally, Vahid et al. (2023) revealed that nutritional metabolomics is a rapidly evolving field developed as an interplay between dietary factors, metabolic changes, and breast carcinogenesis or breast cancer risk (BCR) [103]. Nutritional epigenomics/epitranscriptomics/epiproteomics are focused on the influence of nutrients and bioactive dietary components on cytosine methylation, histone PTMs and specific RNA molecules as main epigenetic factors involved in BC development [104].

It was demonstrated that a wide variety of environmental xenobiotics, including persistent organic pollutants and endocrine disruptors, act on different pathways involved in invasion and metastatic process in BC (Table 1), such as EMT and stemness maintenance, tumor suppression and induction, estrogen biosynthesis and signaling, DNA methylation, gene transcription, proliferation, or inflammation [92,94]. Feng et al. (2018) summarized many environmental risk factors involved in BC development and progression, such as exposure to diethylstilbestrol (DES), lifestyle-related risk factors, contraceptives, hormone replacement therapy (HRT) after menopause, excessive alcohol consumption, dietary habits and obesity, or lack of physical activity [93]. Terry et al. (2019) showed that the influence of environmental chemicals on BC risk may be grater during distinct time periods in a woman’s life, such as prenatal development, puberty, pregnancy, and perimenopause, known as windows of susceptibility (WOS), because of significant structural and functional changes that affect the mammary microenvironment and hormonal signaling pathways [94].

Alcohol consumption confers a high risk for BC development and its effects seem to be stronger among estrogen receptor (ER)-positive tumors [105]. Alcohol stimulates migration and invasion in MCF7 human BC cells [105], EMT, angiogenesis, oxidative stress (OS) and reactive oxygen species production (ROS) [106,107], decreasing the expression of E-cadherin, α, β, and γ catenin, and BRCA1 tumor suppressor gene [105]. Also, alcohol regulates several genes that are associated with the response to endocrine therapy and attenuates the action of tamoxifen in BC cells [108]. On the contrary, coffee is a cocktail of more than 1000 described phytochemicals with anti-tumor, anti-inflammatory, and anti-oxidant proprieties [109]. Both green or dark coffee, as well as green or black tea, are associated with decreased BC risk [110,111]. Coffee consumption might reveal chemopreventive and chemotherapeutic effects, coffee extracts emphasizing anticancer activity by reduction in viability and proliferation in MDA-MB-231 (ER–) and MCF7 (ER+) BC cell lines [112]. Also, these extracts demonstrated the anti-tumor effects of freeze-dried robusta coffee (*Coffea canephora*) [112]. Several compounds, such as coffee chlorogenic acid (CGA), which is also found in blueberries, plums, and cherries [113], exerts an inhibitory role on the NF-κB/EMT signaling pathway, viability, migration and invasion in BC cell lines, as well as a pro-apoptotic role [114]. Caffeine enhanced the cisplatin treatment activity in TNBC MDA-MB-231 and MCF7 cell lines [115]. Green tea epigallocatchin-3-gallate (EGCG) is also known to significantly reduce BC risk by decreasing ROS as well as oxidative DNA damage, mutagenesis, and tumor progression, or inducing apoptosis in MCF7 BC cell line [111]. Dietary compounds are considered epigenetic modulating agents in cancer [113]. Resveratrol (RVT), one of the most studied AT molecules, inhibits mitochondrial respiration and exerts cytotoxic effects through the stimulation of sirtuin 1 and 3 (SIRT1/3) that reduce the stemness markers in BC cells [116]. Piperine, an alkaloid found in black paper (*Piper nigrum*), inhibits the growth of human BC cells and xenografts in immune-deficient mice, cell cycle progression, matrix metalloproteinases 2 and 9 (MMP2 and MMP9) mRNA expression, BC cell migration, and induces caspase-dependent apoptosis via mitochondrial pathway [117]. Carotenoids, lipophilic micronutrients in fruit and vegetables, which play a role in photosynthesis, have been associated in human plasma with several metabolites involved in immune regulation (tryptophan), redox balance (plasmalogens, glutamine), membrane signaling, epigenetic regulations (acetylated/methylated metabolites), and β-oxidation (carnitines) [118]. Table 1 summarizes several effects of environmental exposure on BC cells and molecular mechanisms involved in BC tumorigenesis and progression, highlighting several omics-based analyses that drive the exposomics approaches.

**Table 1:** Biomolecular effects of several environmental exposures on BC tumorigenesis and progression.

Environmental Exposure	Relevance in BC	Effects on BC Cells and Molecular Mechanisms	Materials	Methods for Biomarkers Investigation
Exogenous hormones and EDCs				
Omics: transcriptomics [119,120,121], exposomics and epigenomics [122,123], blood proteomics [124], organoid proteomics [125], metabolomics [126]				
Postmenopausal HRT/oral contraceptives/hormone treatment/endocrine therapy	increase of 20–30% in BCR associated with current or recent use of either oral combined or progestagen-only contraceptives [127]	increases epithelial proliferation in postmenopausal TDLUs [128]	benign breast biopsies [128]	IHC, comparative breast epithelial density [128]
Bisphenols (BPA, BPAF, BPF, BPS)	increased BCR in mice [129]	BC cell growth, proliferation and migration, activation of signal transduction pathways (STAT3, PI3K/AKT, GPER/EGFR/ERK1/2; MEK/ERK), epigenetic silencing of tumor suppressor genes, apoptosis, OS, glucose metabolism, angiogenesis, resistance to endocrine therapy [119,122,130]; in utero BPA exposure alters the stroma to increase ECM density and mammary gland stiffness [120]; BPA alter the proteolysis and isoform expression by alternative splicing [125]	hBC cell lines, animal models, clinical studies, human blood samples, mouse mammary organoids [119,124,125]	RT-qPCR, ChIP-qPCR [119], TMT-MS [124], LC-MS/MS [125], RNA-seq profiling of adult primary fibroblasts, SHIM and collagen fiber analysis [120]
Diethylstilbestrol (DES)	increased BCR in pregnant women, daughters, and granddaughters [123,131]	deregulation of mammary gland differentiation and development-related genes may be induced and cause the increased number of TDLUs in human mammary glands [132]; epigenetic alterations: increased DNA methylation, modification in histones and miRNA expression [123]	animal models/tissue samples [132]	RT-qPCR, IF [132]
Phthalates (MEHP, MBzP, DEHP, DBP) and phthalates substitutes (ATBC)	PT [126], high-level DBP exposure associated with 2-fold increase in the rate of ER^+^-BC [133]	ATBC may be involved in cell proliferation [126]; promotes BC cells growth through ER signaling [133]	human plasma samples [126], mice mammary organoid cultures [125], cohort studies [133,134]	TMT-MS [124], LC-MS [126]
Parabens (MP, EP, PP, BP)	earlier BC development [135], potent carcinogens [121]	mimic of endogenous hormones, interact with signaling transduction pathways, such as HER2 signaling, modulate of key enzymes involved in estrogen metabolism, increase pro-oncogenic c-Myc expression in ER+/HER2+ BC cells, promote EMT [135]	BC and non-malignant cell lines [121]	RT-qPCR, WB [121]
Polychlorinated biphenyls (PCBs)	increased BCR [136]	as EDC [137], enhance metastatic proprieties of BC cells by activating ROCK, increase cell motility/migration/invasion, disease progression, induce the intracellular ROS production [138]	human BC cell lines, animal models [138], mice mammary organoid cultures [125]	LC-MS/MS [125]
Dioxins and dioxin-like chemicals (TCDD)	controversially role: positive association between airborne dioxins and invasive BCR [139]; no increased BCR for long-term airborne dioxins [140]; no association between dietary dioxin and BCR [141]; significant positive association between dioxin exposure and BCR [142]; protective effect against BC [143]	disruption of the CXCL12/CXCR4 axis limits the metastasis of BC cells to the lung in mice [143]; BC cells may acquire pro-metastatic and CSCs features [144]	BC cell lines [143], co-culture model using MCF7 and MDA-MB-231 BC cell lines together with hMADS preadipocytes and in vivo Zebrafish larvae model [144]	gene chip microarray, RT-PCR, FC [143], nLC-MS/MS, qRT-PCR, Zebrafish larva metastasis assays [144]
Dietary factors [145]				
Omics: nutrigenomics and nutriproteomics [102], nutritional epigenomics/epitranscriptomics, and epiproteomics [104], phosphoproteomics [146], metabolomics/nutritional metabolomics [103,145,146]				
High fructose intake	PT [147,148], fructose-induced carcinogenesis [146]	metabolic reprogramming, uncontrolled BC cells growth, apoptosis inhibitor in TNBC [147]; increasing in colony formation and migratory capacity of BC cells, GLUT5 overexpressed in BC cells and tumor tissue but not in normal counterparts [148], promotes Warburg effect [149], and triggers BC cells proliferation and metastasis/invasion through ketohexokinase-A (KHK-A) signaling pathway [146]	BC cell lines, animal models, BC xenografts [146,148]	qRT-PCR, ChIP, immunoblotting and immunoprecipitation, IF, IHC, LC-MS/MS to identify PTMs, GC-MS to identify metabolites, hBC tissue microarray [146]
Dietary phytoestrogens (i.e., from soy beans: genistein/GNT, and daidzein)	controversial role: associated with lower BCR [150], potent AT agents [151], stimulate proliferation of ERα+ BC cells at low concentrations [145]	mediates the AT mechanisms through apoptosis induction, arresting cell cycle, inhibiting angiogenesis and metastasis, mammosphere formation, targeting and suppressing tumor growth factors, upregulating tumor suppressor genes and downregulating oncogenes [151,152]	BC cell lines [151]	LC-HRMS to identify estrogen metabolites [145]
Commonly used spices	AT [117]	piperine inhibits growth of BC cells and xenografts in immune-deficient mice, cell cycle progression, MMP2 & MMP9 mRNA expression, BC cell migration, and induces caspase-dependent apoptosis via mitochondrial pathway [117]	animal models, BC cell lines (MDA-MB-231, MCF-7) [117]	flow cytometry, western blot, qRT-PCR [117]
Alcohol consumption	PT	stimulates BC cells mobility, EMT, cell adhesion, migration and invasion, angiogenesis, OS, ROS production, proliferation of ERα+ BC cells in vitro; decreases E-cadherin, α, β, and γ catenin and BRCA1 expression; alteration in methylation pathways [105,106,107,153,154]	BC cell lines	WB, Illumina bead chip arrays, qPCR
Coffee and tea products	no association between overall coffee drinking and BCR or slightly protective effect [155]; positive association of instant coffee consumption with BCR [156]; AT effects of tea compounds [111]	CGA-inhibitor of NF-κB/EMT signaling pathway, viability, migration and invasion in BC cells, pro-apoptotic role (AT) [114]; caffeine enhanced the cisplatin treatment activity in TNBC MDA-MB-231 and MCF7 cell line [115]; EGCG induces apoptosis in MCF7 cells [111]	BC cell lines	FLIM [115]
Resveratrol	AT [116,157]	activator of BRCA1, p53, p21, PRMT5 and EZH2 inhibitor [157]; SIRT activator [116]	BC cell lines [116,157]	WB [116]

Abbreviations: AT-anti-tumor; ATBC-acetyl tributyl citrate; BCR-breast cancer risk; BP-butylparaben; BPA-bisphenol A; BPAF-bisphenol AF; BPF-bisphenol F; BPS-bisphenol S; CGA-chlorogenic acid; ChIP-chromatin immunoprecipitation; Cu-cooper; DBP-dibutyl phthalate; DEHP-di(2-ethylhexyl) phthalate; ECM-extracellular matrix; EDC-endocrine disrupting chemicals; EGCG-epigallocatchin-3-gallate; EMT-epithelial-mesenchymal transition; EP-ethylparaben; EZH2-enhancer of Zeste hololog 2; FA-fatty acids; FC-flow cytometry; FLIM-fluorescence lifetime imaging microscopy; GC-MS-gas chromatography mass spectrometry; G47Δ-mIL12-herpex simplex virus encoding an anti-tumor cytokine, IL-12; HCMV-human cytomegalovirus; HPV-human papilloma virus; HRT-hormone replacements therapy; IF-immunofluorescence; IHC-immunohistochemistry; LC-HRMS-liquid chromatography-high resolution mass spectrometry; MBzP-mono-benzyl phthalate; MEHP-mono-2-ethylhexylphthalate; MGMT-O6-methylguanine-DNA methyltransferase; miRNA-microRNA; MMTV-mouse mammary tumor virus; MP-methylparaben; MT-metallothioneins; OS-oxidative stress; PCNA-proliferating cell nuclear antigen; PP-propylparaben; PRMT5-protein arginine methyltransferase 5; PT-pro-tumorigenic; PTMs-posttranslational modifications; qRT-PCR-reverse transcription quantitative PCR; ROCK-Rho-associated kinase; ROS-reactive oxygen species; SFA-saturated fatty acids; SHIM-second-harmonic imaging microscopy; SIRT-sirtuin; TCDD-2,3,7,8-tertachlorodibenzo-*p*-dioxin; TDLUs-terminal duct-lobular units; TNBC-triple negative breast cancer; TMT-MS-tandem mass stag and quantitative mass spectrometry.

Following another perspective, BC has often been characterized by specific terms that belong to ecology. Population, species, community, and ecosystems are known as concepts of ecological units that are at the basis of ecological theory [158]. Hirpara et al. (2018) sustained that carcinogenesis is a form of speciation, because cancer cells share several characteristics with conventional species: autonomy, karyotype individuality, immortality, and long latency from carcinogens to cancer initiation and progression [159]. Tumors consist of heterogeneous populations of cancer cells, known as clones/subpopulations, within a stroma with different normal and tumor/cancer-associated cells (TACs/CACs), interacting each other and their extracellular matrix (ECM), defining a specific tumor microenvironment (TME) that form together the “tumoral ecosystem” [160]. An ecosystem comprised of biotic community and non-biotic environment, while the tumoral ecosystem comprised of interacting cancer and non-cancer cell populations and their ECM. Cancer cells adopt phenotypic strategies to gain access to and invade novel territories through cell-extrinsic degradation of ECM barriers for exploitation of local resources, while the ecological succession allows for the emergence of large, heterogeneous, resistant and highly proliferative subpopulations of tumor cells [161]. Korolev et al. (2014) suggested that the cancer cells can be viewed as an endangered species, in order to exploit cancer vulnerabilities to drive its extinction, using the ecology of tumors for treatment [162]. Gregg (2021) combined knowledge from ecology, evolution, biochemistry, infectious disease, species extinction, metabolism, genomics and epigenetics to develop clinically relevant strategies to constrain cancer cell diversity and adaptability to enhance treatment efficacy in metastatic cancer by association of treatment with ecological factors, such as hyperthermia, fasting, and immunotherapy, similar to starvation and climate change in nature as factors that drive the extinction of species [163].

Comparisons between cancer and ecological systems are extensively reviewed [164]. Thus, tumors express proprieties of ecological systems, so that many authors proposed the theory of tumoral ecosystems, which analyzes BC as a metabolic ecosystem or an immune ecosystem [165,166,167], or reveals the BC ecosystem diversity, dissecting the ecosystem cellular composition, morphology, spatial organization, and the relationship between various cell types [168]. Also, the ecological therapy for cancer define and monitor the treatment response based on this ecosystem paradigm [169]. Kotler and Brown (2020) adopted five major categories of mechanisms of coexistence of cancer cell species that form local ecological communities within tumors: food-safety tradeoffs, diet choice, habitat selection, variance partitioning, and competition-colonization tradeoffs [170]. Multi-omics-based approaches enlighten the complexity and the heterogeneity of BC ecosystem by integrating the multidimensional characteristics, while the eco-oncology may improve the understanding of the BC biology, the role of molecular biomarkers and principles of evolutionary medicine [87,166,171]. Thus, the similarities between: (i) heterogeneous cancer cells interacting with various cellular or non-cellular components in the host internal environment and living organisms interacting with each other and their environment, (ii) initiation and growth of tumors and dynamics of biological populations, and (iii) between metastasis and ecological invasion or communities dynamics, lead to an ecological perspective to improve BC biology and therapy [87,165].

### 3.4. BC Is an Evolutionary Disorder

The evolution of a species is a long-term process that shapes their neoplastic profiles [172]. Evidence showed that oncogenic tumors date back approximately 2 million years ago [95]. Written records and illustration of BC date back to antiquity (3000–2500 B.C.) [173], a diagnosis of metastatic carcinoma secondary to an unknown soft tissue cancer being dated in 1200 B.C., by using a taphonomic radiographic, microscopic, and scanning electron microscopic imaging of the osteolytic lesions [174]. To understand the long-term cancer evolution, onco-paleogenomics could emerge as a consequence of the recent progress in whole-genome sequencing (WGS) that leads to the reconstruction of the ancestral pathogenomes [175]. Moreover, onco-phylogenomics was born at the interface between biomolecular sciences and evolutionary history of tumors [176], including the evolution of BC that affects most mammal species [177]. Additionally, in 2022, Svante Pääbo was awarded the Nobel Prize in Physiology or Medicine, for opening the way to the development of field of paleogenomics, which can improve the current understanding of modern humans, their ancestors and human evolution [178], also deciphering the genetic evolution of diseases in humans, based on ancient DNA (aDNA) analysis through modern genomics-based methods [179]. Advances in paleogenomics-based medicine may highlight the evolution of predisposition to cancer disease by the identification and characterization of germline mutations in tumor suppressor genes in ancient human genomes [56].

At the organismal level, cancer initiation and progression are also considered as short-term evolutionary processes, in which cells acquire transformative, proliferative and metastatic capabilities and are subject to selection promoted by surrounding tissue ecology of the restrictive niches in the body, such as the site of the primary tumor, circulatory system, and diverse metastatic sites [29,180]. Thus, BC could be characterized as an “ecosystem” of co-evolving clones, competing and cooperating with each other and with cancer-associated cells, the TME becoming an arena of competition, predation, parasitism and mutualism [23]. BC acquires a populational structure, reflected by intratumoral heterogeneity; thus, tumorigenesis becomes an evolutionary process driven by mutations and clonal expansions [181]. These subsequent genetic and selection processes in tumor allow cells to lose their initial tissue identity and migrate to other organs, resulting in cancer dissemination [172], changing environmental conditions, and certainly altering tissue and tumor features [180]. Consequently, the tumor progression might be viewed as a Darwinian process, where the evolution of cancer into the body from a common metastatic precursor might be compared to the speciation, known as the origin of new species from an ancient common ancestor organism [182]. Similarly to populations living in different ecological environments undergo evolutionary changes through divergent natural selection, the TME plays a functional role as a selective pressure that actively participates in cancer progression [182]. Tumor cells are highly constrained by the TME to phenotypically converge, to survive and reproduce into a complex eco-evolutionary system, to feed, avoid predation, migrate, and construct adaptive niches [183].

Many studies treated cancer cells as individuals of a non-sexually reproducing species, as long as BC cells successively substitute in a tumor and the significant reproduction of only some cancer cell lines may confer high adaptability to this cancer [184]. Recent studies established branched evolution as a feature of cancer [185] and compares the evolution of cancer, including BC, in the human body to the origin of new species from a common ancestor organism, according to Darwin’s theory [182]. The eco-evolutionary interactions of circulating tumoral cells (CTCs) with the distant tissues governing metastases formation, so that the control of these small cancer populations could be similar to the ”eco-evolutionary rescue” in natural extinctions [186].

Similarities between microbial and cancer cells, phylostratigraphy and the Serial Atavism Model (SAM) of cancer proposed by Lineweaver et al. (2021) converge towards the hypothesis that cancer cells can reactivate an ancestral genetic program that allows them to adopt a series of multicellular-to-unicellular life reversions [187]. Thus, cancer cells reactivate genes of unicellular origin that confer them the ability to adopt a “selfish” mode of life, to survive within toxic and high stressful environments comparable to ancient environments, primitively proliferate, de-differentiate, de-specialize, de-construct, migrate, invade, re-colonize other niches and to recapitulate early forms of life [188]. Consequently, the genes associated with unicellularity are upregulated, while the genes associated with multicellularity, such as adhesion/junction organization, extracellular matrix, and chromosome organization are downregulated in cancer, that favor acquisition of cellular phenotypes able to survive in primitive and harmful conditions [189].

### 3.5. BC Is a Hyphenated Eco-Evo-Devo Disease

It is now known that the eco-evolutionary theories are essential for understanding cancer development at species level, as long as a species evolves by mutation and selection that acts into a population akin to a tumor that develops by somatic mutations and clonal selection of cells within a tissue [190]. If we consider the evolutionary genomics focused on genetic variation between individuals and among populations as an essential determinant of the evolutionary features of a species [191], the somatic evolutionary genomics is focused on genetic mosaicism that occurs during an organism’s development, providing new opportunities to study the origin and progression of cancer [192]. Recently, the evolutionary proteomics integrates more and more the analysis of proteins and their functions in evolutionary biology [193]. In this context, parallels between cancer and ecological systems have been studied and reviewed by many researchers [87,164,165,183,194,195]. Furthermore, Boddy (2022) suggested that evolution and ecology may unify cancer research that requires more than a cellular or molecular perspective [195].

In nature, ecosystems are thermodynamic systems, open to energy and matter that self-organize or self-regulate towards higher complexity and organization, create order, and self-maintain far from thermodynamic equilibrium [196]. Considering the structure and behavior of cancer cells, a tumor may also be viewed as an open and non-linear dynamic system in the non-equilibrium thermodynamic state, self-organizing in time and space, exhibiting high complexity, robustness, and adaptability [197]. In this context, the eco-oncology is focused on tumors as complex, adaptive, and evolving systems, the eco-evolutionary approach helping to understand cancer dynamics, biology, and management [87].

Many mechanisms of cancer progression may be explained by the principles of ecology [198], developmental biology [83,199], and evolutionary paradigms [200,201]. Many authors have discussed the ecological, developmental, or evolutionary strategies for more successful anti-cancer therapies [165], or to understand the ecological and evolutionary bases of cancer vulnerabilities [201]. In this context, cancers can be viewed as pseudo-organs [183,202], as “living organisms” able to build “self-sustainable tumor ecosystems” [165], as well as both genetically and phenotypically heterogeneous populations within individuals and different species between individuals [201], or like local ecological communities that possess limited number of species, according to local conditions [170]. Cancer is considered a by-product of multicellularity, so that many authors have emphasized that cancer progression is a complex eco-evolutionary process, suggesting that understanding cancer’s evolutionary history may effectively help to manage and treat cancer [203].

Tot (2005–2008) launched the theory of “the sick breast lobe” [204,205,206], integrating genomic, embryologic, and ecological factors that drive the BC development. According to this hypothesis, the majority of breast malignant tumors are considered as “lobar diseases”, because the BC development, as a “long-life” process, arises into a single sick breast lobe that emphasizes a certain degree of genetic instability acquired through mutations during embryonic development, followed by successive accumulation of genetic and epigenetic changes that occur during postnatal lifespan due to exposure to environmental xenobiotics/noxes [207]. Moreover, Tan and Tot (2018) showed that both the ductal carcinoma in situ (DCIS), as well as the lobular carcinoma in situ (LCIS), follow the catchment patterns of breast ductal theories [207]. Thus, BC development as a lobar disease could be compared with the integrated catchment-lake ecological models, which postulate that a natural headwater catchment is the fundamental unit that connect the land to the ocean [208], taking into account that altered patterns into a lake catchment lead to modifications in the lake ecosystem itself [209].

Phylogenetic biogeography is usually used to reconstruct the evolutionary relationships of species and to trace their origin and past geographic distribution, but, recently, tumor biogeography has been introduced to reconstruct evolutionary relationships, genetic divergences, extinction of cancer cells events, and cancer cell migration strategies [210]. Chroni and Kumar (2021) proposed that tumors can be considered evolutionary island-like ecosystems that undergo evolutionary and spatio-temporal dynamic processes that shape tumor microenvironment (TME) and drive cancer cell migration [211]. Gatenby et al. (2020) hypothesized that large, diverse, and disseminated cancer cell populations could be eradicated using similar eco-evolutionary dynamics of Anthropocene species extinctions as an alternative model for cancer treatment [212]. Drawing parallels and contrasts between dormancy in cancer and other ecosystems in nature, Miller et al. (2021) emphasized the potential for studies in cancer to provide insights into the evolutionary ecology of dormancy [213]. Gatenbee et al. (2020) used species distribution modeling (SDM) or histoecology-based models to identify critical environmental factors that drive tumor development and predict response to therapy [214]. Additionally, cancers have been compared with invasive species in terms of invasion, growth, spread, treatment, and outcome, targeting the development of novel paradigms to cure cancer [215,216]. The concept of invasion is widely used in tumor biology, as well as in ecosystems science, providing opportunities to study the mechanisms of invasion at the molecular level [216]. Thus, high-throughput proteomics alone, or in association with complementary transcriptomics and genomics-based approaches, has the ability to identify multiple biomarkers of tumor invasiveness [217]. Multi-omics data, including gene expression, protein expression, miRNA, DNA methylation, and somatic mutation, may provide a comprehensive characterization of invasiveness-related molecular features across multiple cancer types [218]. Moreover, accumulating evidence showed that tumors act like parasites that fight to survive and optimize their fitness inside the host’s body ecosystem [164]. Experimental evidence suggests that some periods of the day are better that other for cancer proliferation and speed, because the neoplastic cells could exploit or manipulate the host biological rhythms [219]. Thus, tumor development has been compared to infectious disease progression [220].

Also, among other cancer types, BC develops through a process of somatic evolution and arises through natural selection at the cell level [28]. Consequently, cancer has been described as a process of Darwinian evolution [201], the differences and similarities between evolution among somatic cells versus evolution among organisms is a rising discussion [221]. As a clonal evolutionary process, BC may be caused by successive accumulation of genetic alterations followed by clone expansion that leads to tumorigenesis, progression, dissemination, and treatment resistance [32]. Moreover, the mutated tumor cells seem to adapt to microenvironmental niches called a tumor microenvironment (TME) better than normal cells, following different gene–TME interaction patterns that involve the history of genetic and epigenetic changes of the cells and the challenging TME characteristics [222]. Like many species in nature and according to the evolutionary fitness paradigm, cancer cells are constrained by their environments to adopt ecologically driven convergent phenotypes, known as cancer hallmarks, which ensure their fitness within the ecological conditions from body tissues [183]. Like in nature, TME condition includes both abiotic (i.e., local temperature, oxygen partial pressure, nutrient supply, blood flow, pH, intercellular fluid biochemistry) and biotic factors (tumor cell phenotypes, stromal cells, immune cells, vasculature) that interact with heritable features of cancer cells. Evolution by natural selection favors some clonal “species” over others, favoring certain subpopulations of cells that possess genetic and epigenetic traits, which allow for their high proliferation and immune evasion [223]. Advances in new spatial genomics-, transcriptomics- and proteomics-based methods offer new opportunities to study cancer evolution with molecular and spatial detail, for defining the main patterns in which cancer and its TME co-evolve [224].

Consequently, the eco-evolutionary theories are essential for understanding cancer development. Herein, we used the integrated framework of three well known ecological theories: the Bronfenbrenner’s theory of human development, the Vannote’s River Continuum Concept (RCC), and the Ecological Evolutionary Developmental Biology (Eco–Evo–Devo) theory, to explain and understand several eco-evo-devo-based principles that govern BC progression.

### 3.6. Can Ecological Evolutionary Developmental Biology (Eco-Evo-Devo) Theory Be Applied to BC Development?

The Ecological Evolutionary Developmental Biology (Eco-Evo-Devo) theory has been conceived to explain the complex interaction between an organism’s environment, genes, and developmental processes [225,226]. Initially, this theory had a major goal to demonstrate how the developmental pathways integrate environmental inputs, generating environment-dependent phenotypes [227]. Cancer development within an individual shapes the species evolution and, in turn, species evolution shapes the neoplastic profile of the species as well [190]. Plowman and Plowman (2021) showed that onco-ontogeny recapitulates phylogeny, such that the Haeckel’s controversial biogenetic law emphasizes a certain relevance to cancer behavior [228]. More recently, Liu et al. (2021) presented a framework of a novel theory titled Cancer Evolution-Development (Cancer Evo-Dev), referring to the inflammation-related hepatocarcinogenesis that is sustained by the interaction between genetic predispositions and environmental exposures [229]. Kozlov (2023), considering three major types of biological development—individual, evolutionary, and neoplastic—showed that the Carcino-Evo-Devo theory sustains the evolutionary role of heritable tumors in the evolution of multicellular organisms [230]. Thomas et al. (2017) hypothesized that, apart from related microbiota and parasites, multicellular organisms have a long evolutionary history with communities of cancer cells, known as oncobiota, that influences host life-history traits and survival strategies [231]. Herein, we used the integrated framework of two well-known ecological theories: the Bronfenbrenner’s theory of human development and the Vannote’s River Continuum Concept (RCC), to better understand several eco-evo-devo-based principles that govern BC progression.

### 3.7. Can Bronfenbrenner’s Theory Be Applied to BC Development?

**Figure 1:**
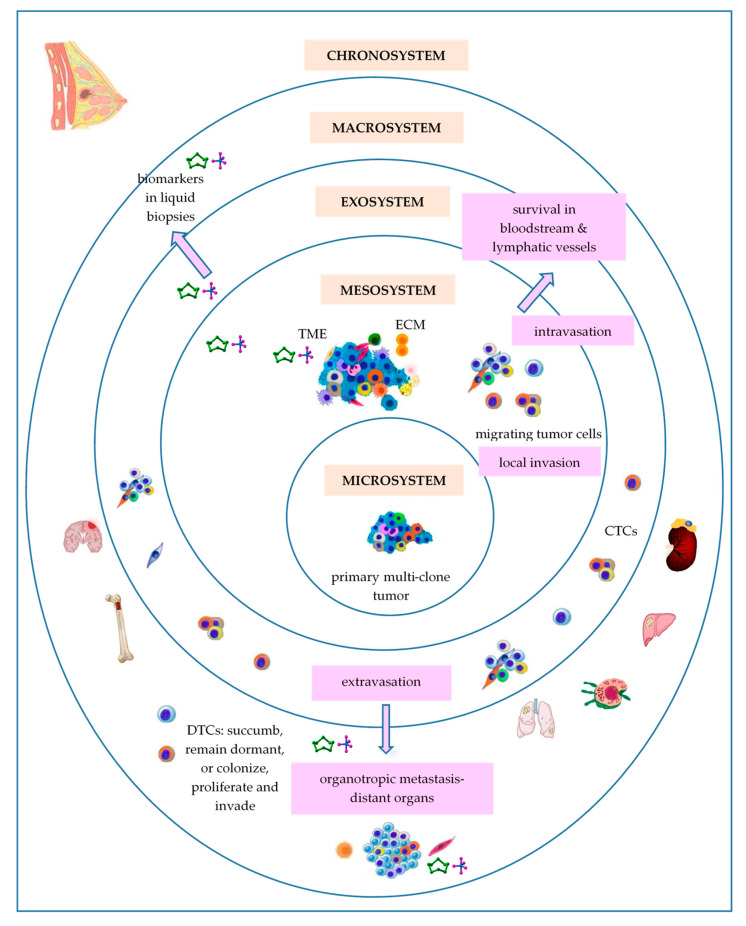
Bronfenbrenner’s theory applied to BC development. Abbreviations: CTCs—circulating tumor cells; DTCs—disseminated tumor cells; ECM—extracellular matrix; TME—tumor microenvironment.

Bronfenbrenner’s theory of human development was first proposed in the 1970s and has been modified over time, evolving from an ecological to a bio-ecological approach [232]. In the BC field, adaptations of Bronfenbrenner’s ecological systems theory have been used in order to assess the role of personal characteristics and needs, dynamic influences of time, interaction with family and health professionals, palliative care services/systems and societal influences involved in BC monitoring and management at a social level [233]. Bronfenbrenner’s ecological systems theory sustains that individual system development is influenced by a series of interconnected environmental systems (Figure 1), such as the microsystem, mesosystem, exosystem, macrosystem, and chronosystem [232].

#### 3.7.1. Primary Breast Tumor as a Microsystem

Considering BC cells of origin as a central ring of Bronfenbrenner’s model, the microsystem is the most proximal setting, with particular physical characteristics, in which these cells are situated. Like all living systems, BC cells of origin are able to self-renew [234]. According to the original theory of cancer stem cells (CSCs), BC, as well as most solid tumors, may originate in a unique cell named a breast cancer stem cell (BCSC), due to an aberrant genetic event that has occurred into a mammary stem cell (MaSC) or within a progenitor cell, even long before the cancer diagnosis. Further, BCSCs retain the immortal proprieties of MaSCs throughout tumorigenesis. BCSCs, also called tumor-initiating cells (TICs), are capable of long-term self-renewal and differentiation, and may divide symmetrically or asymmetrically [235]. Finally, BCSCs account for only 0.1–1% of all tumor cells, representing a minor but significant subpopulation of undifferentiated cells in a tumor [236]. The clonal evolution model sustains that BCSCs might arise through the reprogramming of differentiated cells of mammary tissue, such as luminal and myoepithelial cells, which emphasize a specific mammary mutational profile, suffer a de-differentiation process, and regain stem-like proprieties that lead to new generations of BCSCs. Apart from the auto/self-renewal and stemness capacity, BCSCs have clonal tumor initiation capacity and clonal long-term repopulation potential sustained by specific self-renewal signaling pathways (SRPs) that are commonly dysregulated due to genomic and epigenomic changes [237,238]. Heyde et al. (2019) showed that intra-tumoral heterogeneity emerged as an inevitable consequence of cancer development so that many clones coexist in primary tumor at the time of cancer diagnosis [239]. Additionally, distinct from this classical linear pattern for the evolution of a unique cancer founder, a branching model of evolution of multiple cancer founders may be possible during cancer development [240]. Then, these related clones dissipate and invade the local environment and expand along mammary ducts and occupy a large area in the breast [240].

All living systems have, as a general feature, a hierarchical organization [241]. The theory of CSCs suggests that there is a hierarchy in cancer cells, in which CSCs lie at the top level [235]. Cole et al. (2020) showed that many heterogenic cancers, including BC, are organized into hierarchical structures, based on differentiation capacity of CSCs, the differentiation hierarchy model of cancer being dependent on the gain and loss of the different biomarkers used to characterize the different CSCs populations [242]. Moreover, a continuum of states from stem to differentiated cells exists, enhancing their adaptability and emerging in difficulties for cancer therapy [235].

Competition is a critical evolutionary mechanism that influences the spatio-temporal and dynamic pattern and persistence of species [243]. Tissues and organs may be considered as “social groups” governed by “societal rules” that allow for normal cells to cooperate, while mutant cells compete and expand their territory with the biomolecular aid of their neighboring non-tumor cells [244]. Some authors have claimed that tumors can be viewed as an “invasive species” [215], which express intelligent behavior [245]. As a species is composed by multiple populations, a tumor is composed by multiple clones. Some of these cells move from primary to distant metastatic sites and develop multiple adaptation strategies to survive in hostile environmental conditions. Madan et al. (2022) showed that cell–cell and cancer–microenvironment competition may be a key mediator of clonal dynamics throughout the neoplastic process, being involved in tumor initiation, field cancerization, tumor outgrowth at the expense of normal tissue, and development of malignant cancers from premalignant lesions [246]. Desjardins-Lecavalier et al. (2023) used a method based on single-cell magneto-optical capture (scMOCa) to isolate fast cells with a highly migratory phenotype from heterogeneous human BC cell populations, exploiting their migratory ability alone [247]. Moreover, the fast cell subpopulations expressed genes associated with cell migration, generated a high number of CTCs and soft tissue metastases in mouse models, and retain their high migration speed and focal adhesion dynamic over many generations due to their motility-related transcriptomic profile [247].

As any other living systems, BC microsystems emphasize the capacity to adapt and cope, according to adaptive capacity of complex ecosystems [248,249]. Tumor cells have the ability to reprogram their phenotype, in terms of shape, structure, metabolism, and behavior, in order to cope/adapt with/to environmental challenges, such as local hypoxia, acidity, local temperature, low nutrient supply, or chemotherapy [250]. For example, the oxygen content of primary tumor tissue is an inductor for metastatic cascade [251]. The mean partial pressure of oxygen (PO_2_) inside the breast tumors ranges from 2.5 to 28 mm of mercury (Hg) (usually < 0.1–5% in solid tumors [252]), with a median value of 10 mm Hg, as compared with 65 mm Hg in normal breast tissue [251]. A starvation-pseudostarvation model suggests that metastasis may be induced by starvation due to oxygen or nutrient limitations that cannot sustain the cell growth and proliferation, or by pseudostarvation imposed by oncogenic activation or microenvironmental signals that converge in translation reprogramming [253]. Lozano et al. (2020) developed a computational thermal model of BC, showing that the metabolic heat generation rates reached as high as 20,000 W/m^3^ for normal breast tissue and ranged between 100,000–1,200,000 W/m^3^ for cancerous breast tissue in the case of triple-negative BC (TNBC) [254]. The contact thermography applied to women with primary invasive ductal carcinoma (IDC) showed that the temperature of tumor was 1.79 ± 0.88 °C higher that of the surrounding tissue, in correlation to the microvessels density [255]. Moreover, tumor acidity affects most steps in the metastatic cascade [256]. Cancer cells have increased intracellular pH (pHi) and decreased extracellular pH (pHe) compared to normal cells that also affects cell proliferation, metabolic adaptation, and tumorigenesis by altering the structure and function of pH-sensor proteins [257]. Thus, the pHi ranges from 7.3 to 7.6, while the pHe of solid tumors ranges from 6.5 to 6.9, whereas pH ranges between 7.2 to 7.4 in normal tissues, compelling cancer cells to develop interrelated mechanisms of adaptation to acidity for survival, such as autophagy, increased lysosomal turnover and redistribution, or increased intracellular lipid droplets density [258].

#### 3.7.2. Breast Tumor as a Mesosystem

According to Bronfenbrenner’s ecological systems theory, the mesosystem is the level of complex relationships among two or more microsystems, allowing the microsystem’s development [232].

It is known that tissues provide the context for cancer cell development and progression [259]. Thus, the primary/local tumor microenvironment (TME) may be considered as a mesosystem, in which heterogeneous populations of tumor cells that form the microsystem of the primary tumor interact with other microsystems represented by different types of non-tumor cells, such as infiltrating inflammatory cells (tumor infiltrating lymphocytes (TILs), dendritic cells (DCs), macrophages, and neutrophils) and stromal cells (fibroblasts, myoepithelial cells, adipocytes, endothelial cells, and pericytes), as well as the extracellular matrix (ECM) that includes various soluble and physical factors, such as enzymes, cytokines, growth factors, pH, or oxygen levels, to facilitate tumorigenesis. This interaction plays crucial roles in tumor proliferation, invasion, and therapies response [260]. Hence, the tumor cells modify the niche that they occupy to offer them a selected advantage over heathy cells [164]. Mayer et al. (2023), using single-cell RNA-sequencing (scRNA-seq), demonstrated that the human BC TME shows a hierarchical network structure of cell–cell interactions, dominated by cancer-associated fibroblasts (CAFs) [261], the most abundant and heterogeneous cellular components of the TME [262]. Local normal fibroblasts and mesenchymal cells adapt to the mesosystem local conditions, becoming activated CAFs and myofibroblasts, which reshape the ECM by “dermoplastic reaction” [262]. Moreover, CAFs may be derived from other multiple cell types, such as epithelial cells, endothelial cells, bone marrow-derived mesenchymal cells, transdifferentiated adipocytes, transdifferentiated smooth muscle cells, and resident stem cells, that can form distinct populations in the same stroma [263]. It was also demonstrated that differential oxygenation in TME modulates macrophages, the most abundant immune cell population in the majority of solid tumors, and cancer cell crosstalk [252].

The tumor mesosystem is the place of cell–cell competition, prey–predator and/or host–parasite interactions, and cell migration. Thus, tumor-immune cells interactions may be imperfectly compared with predatory-prey competition in nature [264]. In the tumor mesosystem there is a high competition for space and low resource supply (i.e., oxygen, glucose or other nutrients) that selects for tumor cells to adopt a dispersal-like behavior from the primary site and to colonize distant organs, while spreading slows when the resource supply is high [265]. Chang et al. (2025) showed that glucose consumption by tumor cells metabolically restricts and suppresses T cells functionality and, consequently, the nutrient competition could determine cancer progression [266]. Moreover, according to Taylor et al. (2017), the starved MCF7 BC cells may suffer epigenetic changes that persist across many generations [265]. The Warburg effect leads to an acidic TME that breaks down the ECM and promotes angiogenesis [267]. The interspecific relationships between human and its symbionts and parasites, which have shaped and are still shaping the human genome, are largely involved in evolution of living organisms [268]. Within this framework, some evidence identified a unique microbial community in breast tissue, as well as in breast tumors, that have their own distinct microbial community that may lead to the occurrence and BC development, for example, by different bacterial toxins that cause DNA damage or regulation of local and systemic immune response [269].

#### 3.7.3. Breast Tumor as an Exosystem

The Exosystem represents the third ring of Bronfenbrenner’s ecological model. In tumor progression, the intravasation and circulation of tumor cells as circulating tumor cells (CTCs) into the bloodstream or lymphatic vessels towards colonization of pre-niches in preferentially distant sites after extravasation may be considered as main metastatic processes that define the tumor exosystem functionality. Generally, solid tumors give rise to heterogeneous populations of CTCs that evade and contribute to tumor dissemination and progression. Some authors suggest that only 0.2–2% of the tumor cells could form micrometastasis at distant organs [270], or approximately 0.01% of CTCs infiltrate and eventually colonize distant organs [271].

In nature, species migration requires eco-morphological, eco-physiological and behavioral adaptations and affects ecosystem processes [272]. Malignant cell migration, dissemination and invasion, through individual (single) cell migration (amoeboid and mesenchymal invasion), and collective cell migration, when multiple cells retain cell–cell connections and migrate coordinately, are essential for metastatic disease [273]. Moreover, cancer cells may adapt their motility patterns by reversible mesenchymal-amoeboid/amoeboid-mesenchymal transitions (MAT/AMT) and individual-collective/collective-individual transitions, such as amoeboid-collective/collective-amoeboid transitions (ACT/CAT) and mesenchymal-collective transition/collective-mesenchymal transitions (MCT/CMT) [273]. Once detached from the primary site, CTCs encounter the bloodstream and lymphatic vessels microenvironments, hostile habitats that stimulate them to adopt the best strategies to survive, such as phenotypic adaptation based on their phenotypic plasticity and metabolic reprogramming, adapting to supportive niches, and collective adaptation [245]. Thus, EMT is involved in genesis of CTCs [274]. EMT is also known as an important embryological step in the gastrulation, heart, musculoskeletal system, or peripheral nervous system organogenesis [274]. Into the metastatic cascade, there are two opposite cellular strategies that alter cancer cell shape: the epithelial-to-mesenchymal transition (EMT) and mesenchymal-to-epithelial transition (MET). During EMT, cancer cells lose epithelial specific biomarkers (i.e., E-cadherin, cluster of differentiation CD44v variant isoform), while acquiring mesenchymal biomarkers (i.e., N-cadherin, fibronectin (FNT), vimentin (VIM), smooth muscle actin (α-SMA), β-catenin (CTNNB), CD44s standard isoform) [275]. Inversely, during MET in distant organs, CTCs abandon their mesenchymal phenotype. Moreover, EMT is also associated with a complex metabolic reprogramming based on mutations in metabolic genes, which support the energy requirements of increased motility and growth in harmful environmental conditions [276]. Consequently, the shift between these very different environments is costly, as in the case of the salmon species that spend their first months in freshwater, and then migrate to saltwater for several years before returning to natal rivers to spawn [277]. Nicolazzo et al. (2023) compared the EMT of tumor cells-to-CTCs with shape-shifter birds and their metabolic reprogramming with naked mole-rats, the most hypoxia-tolerant mammal, as “metabolism switchers” [245].

#### 3.7.4. Breast Tumor as a Macrosystem

BC cells preferentially metastasize to several organs, such as bone (30–60%), lung (21–32%), liver (15–32%), brain (4–10%), known as organotropic metastasis [278]. Thus, metastatic BC cells may be considered as “seeds”, while the microenvironment of the metastatic niche may be viewed as the “soil”, so that the metastatic niche formation depends on “seeds-soil” interactions [279]. It is known that organ-specific stromal cells release signaling proteins that induce chemotaxis, the ability to move in the direction of a chemical gradient, causing organotropic metastasis [280]. The metastatic organotropism depends on the subtype of BC, host organ microenvironment, and cell–cell and cell–matrix interactions. Thus, invasive lobular carcinomas (ILCs) has three times more metastases in the peritoneum, gastrointestinal tract, and ovaries comparative to invasive ductal carcinoma (IDC), which “prefers” to metastasize to the lungs, distant lymph nodes, and central nervous system [281]. The “seeds-soil” interactions facilitate: (i) pre-metastatic niche formation under the influence of factors released by cancer cells before their arrival at distant organs; (ii) metastatic niche formation, and (iii) interaction between disseminated tumor cells (DTCs) and local resident cells, assuring the cancer cell survival and formation of metastatic lesions [282]. Heyde et al. (2019) showed that 10 to 150 cells may seed each metastatic pre-niche and only a fraction of genetic diversity into a primary tumor is passed on to metastases [239]. Schrijver et al. (2018) showed that the main BC gene-drivers have been altered in both the primary tumors and their metastases, but they also identified genetic alterations restricted either to the primary tumor or within metastases to metastasis [283]. In the metastatic niche, DTCs form a metastasis or enter in the dormant period, when cancer cells exit the cell cycle, arrest their growth, and become immune to drugs that usually target cells in mitotic division [284]. Dormancy is known as a reversible state of reduced metabolic activity, known as a dynamic mechanism by which the organisms/cells respond to periods of unfavorable environmental/microenvironmental stress, including temperature, low nutrients, or toxins [285]. For example, the bone is considered a particularly hypoxic environment, where the oxygen levels range from <1% to 6% (approximately 7–43 mm Hg), compared with most normal tissues, where oxygen levels ranges between 2% and 9% (14–65 mm Hg) [286]. Moreover, in the bone marrow, the oxygen level ranges between 1% and 4% (7–29 mmHg) [286]. Smoking promotes lung metastasis of BC, because it generates a neutrophil-dependent pulmonary inflammatory microenvironment [279]. In the brain, BC cells which pass through the blood-brain barrier (BBB), interact with astrocytes, and release matrix metalloproteinases (MMPs) that destroy the collagen network, promoting tumor cells growth and development [281].

Dormant DTCs may reactivate after a long period of dormancy and become a source of BC recurrence [287]. Usually, stromal inflammation might reactivate these cells, inducing growth and a mesenchymal phenotype to dormant ER+ cells with an epithelial phenotype [288].

#### 3.7.5. Breast Tumor as a Chronosystem

The chronosystem is the fifth outermost ring in the Bronfenbrenner’s ecological model that contains elements of time. Cancers begin when the first cell undergoes malignant transformation [289]. Various mathematical models have been used to explore the natural progression of BC [290]. Cancers may evolve over variable time frames that range from 1 to 50 years [259]. It is known that cancer cells double 20 to 30 times to reach 1 mm^3^ to 1 cm^3^ [289]. Thomas et al. (2017) showed that, during the organism’s lifespan, the duration of the interaction between the host and its oncobiota can vary from months to years, sometimes decades [231]. Relative to the time of the first cancer-drive mutation, metastasis has been described as a late event in the natural molecular history of cancer [291]. In BC, metastasis can occur early when the primary tumor is <1 mm in diameter, even with 2–4 years prior to diagnosis of the primary tumor [291]. Menes et al. (2015) have reported that the 10-year incidence of a second primary BC was highest in BRCA1 mutation carriers [292]. Moreover, according to both “the sick breast lobe” hypothesis and the “theory of biological timing”, BC development is subjected to biological timing of transformation of a large number of epithelial cells or stem cells of the sick lobe in tumor cell clones after several cell generations in large carcinomas [205]. Thus, the time needed for this transformation, which may be several decades, the number and location of transformed cells, and the differences in their transformation patterns determine the individual morphology and behavior of BC.

### 3.8. Can Vannote’s River Continuum Concept (RCC) Be Applied to BC Development?

**Figure 2:**
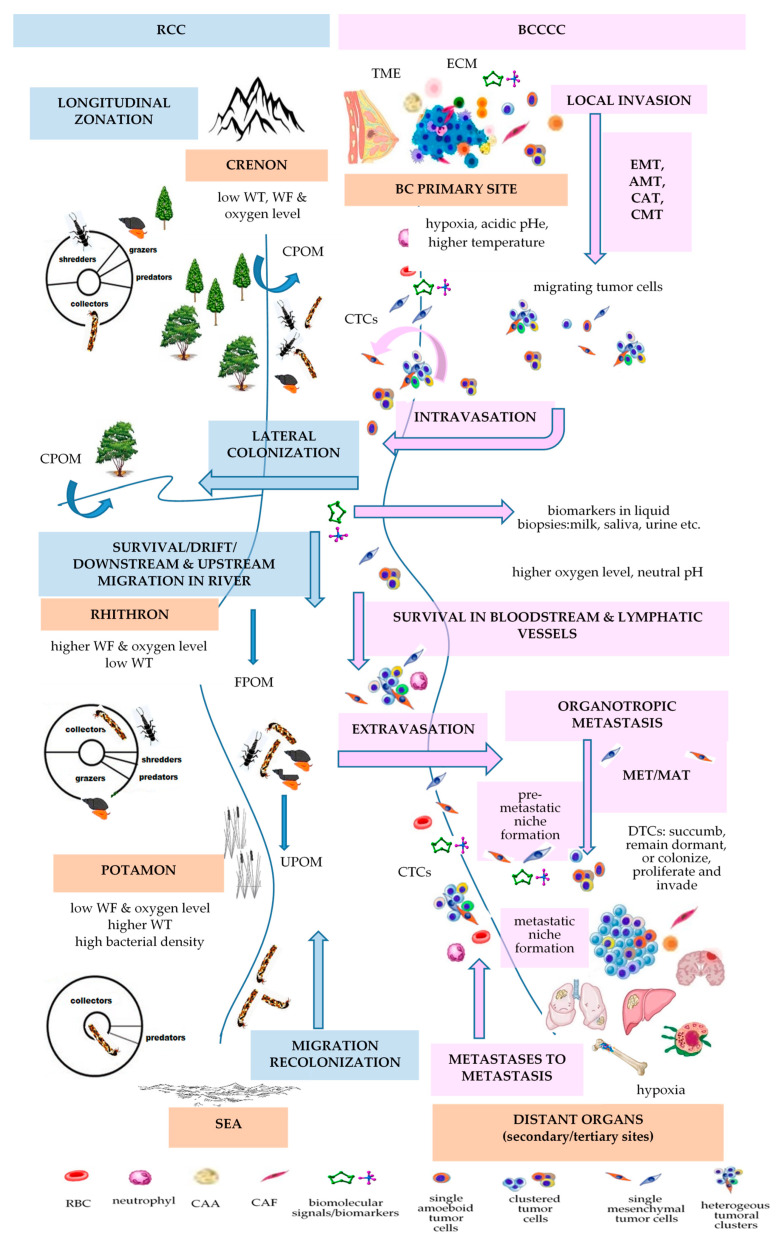
Vannote’s River Continuum Concept (RCC) principles applied to BC development (BCCCC). Abbreviations: AMT—amoeboid-mesenchymal transition; BCCCC—Breast Cancer Cell Continuum Concept; CAA—cancer-associated adipocyte; CAF—cancer-associated fibroblast; CAT—collective-amoeboid transition; CMT—collective-mesenchymal transition; CPOM—coarse particulate organic matter; CTCs—circulating tumor cells; DTCs—disseminated tumor cells; ECM—extracellular matrix; EMT—epithelial-mesenchymal transition; FPOM—fine particulate organic matter; MAT—mesenchymal-amoeboid transition; MET-mesenchymal-to-epithelial transition; pHe—extracellular pH; RBC—red blood cell; RCC—River Continuum Concept; TME—tumor microenvironment; UPOM—ultrafine particulate organic matter; WT—water temperature; WF—water flow.

Chroni and Kumar (2021) categorized tumor ecosystems into islets, islands, and archipelagos-like ecosystems that may transform from one type into another [211]. Here, we aim to discuss cancer progression as a watercourse. The River Continuum Concept (RCC) ecological theory, first developed by Robin L. Vannote et al. in 1980 [293], is considered a “milestone in stream ecology”, due to its comprehensive description and evaluation of the structure and function of river systems [294]. This theory is based on the concept of dynamic equilibrium of a watercourse as an open ecosystem in constant interaction with the bank, moving from headwaters to mouth/sea, and links physical variables with patterns in biodiversity, functionality, and metabolism dynamics, that results in downstream gradients in communities composition and ecosystem processes [294]. Vannote et al. (1980) showed that in natural stream systems, biological communities form a temporal continuum of synchronized species replacements [293]. Comparatively, BC tumorigenesis and metastasis represent a complex cascade/continuum of cells and biomarkers that comprises successively integrated populations of heterogeneous tumor cells and cancer-associated cells, interacting in a dynamic spatio-temporal fashion under specific microenvironmental conditions (Figure 2 and Table 2). Thus, akin to the species of biological communities that form a spatial continuum in a watercourse or in other ecosystem types, cancer cells should have the ability to: (i) move to more hospitable environments when local conditions in primary tumor sites become unfavorable, like in case of hypoxia, nutritional and thermoregulatory stress or acidic pHe, known to promote cell invasion [295]; (ii) move to minimize the cell–cell competition for local resources; (iii) move in biomolecular gradients, following directional migration patterns that may be cell type specific [296] and adjusted by different growth factors and chemokines from the primary TME [297]; (iv) don’t waste effort moving in wrong directions before drifting in the correct direction; and (v) should arrive at a specific distant site in a timely manner based on chemotactic-based behavior that requires sensing, polarizations, and directional motility, emphasizing accuracy, speed and persistence [298]. Moreover, metastasis is a selective process that favors cells with higher deformability and motility [299]. Migrating animals possess ecomorfological adaptations and can often move together, so that collective factors and sensory cues could shape migration [300]. Similarly, increasing evidence suggests that clustered CTCs (microemboli) resist better in circulatory system and emphasize higher metastatic capacities that single CTCs [301]. Migratory animals can adopt adaptive strategies, modifying their behavior, life-history, and physiology through phenotypic plasticity [302]. Similarly, CTCs enter the bloodstream, where they are exposed to immunological insults from leukocytes, to collision with erythrocytes, and to interaction with activated platelets and macrophages, so that only a small fraction of these cells are able to complete the metastatic process, colonizing pre-metastatic niches from distant organs [303]. Kareva (2015) showed that only a small fraction of cells from primary tumors are successful in establishing distant metastasis, a similar process occurs in nature and is known as the ecological succession [164].

Genomics, transcriptomics, proteomics, secretomics or exosomics-based approaches are essential to define the BC continuum (BCC) from primary to metastatic sites. During this metastatic continuum, dissemination of CTCs is a crucial step. CTCs may even assure the “tumor self-seeding”, also colonizing their tumors of origin, where they actively contribute to tumorigenesis [304]. These cells are extremely heterogeneous and form phenotypically and genotypically distinct rare subpopulations of highly active tumor cells released into the bloodstream from primary cancers and metastases that reflect the status of tumor genotypes, as long as mutations in known driver genes found in primary tumor and metastases were also detected in corresponding CTCs together with mutations exclusively observed in CTCs [305,306]. Evidence demonstrates that CTCs undergo modification in response to the dynamic biophysical environment in the bloodstream, partially due to fluid shear stress that generates reactive oxygen species (ROS), which affect mitochondria, causing aberrant energy metabolism, oxidative stress (OS), and cell death pathways that sustain cancer invasiveness [307]. In RCC theory, Vannote showed that the longitudinal distribution and lateral colonization of biotic communities primarily depends on abiotic factor gradients. Similarly, during the metastatic cascade, CTC subpopulations migrate from the hypoxic primary TME to hypoxic pre-metastatic niches (PMN) of metastatic distant sites, through the bloodstream, also considered as a hostile environment for CTCs, where the oxygen level is much higher than in most solid tumors. Proteomics-based techniques have been used to distinguish the proteome landscape of distant metastasis derived from primary breast tumors [308], to identify circulating proteins from serum or plasma, taking into account that proteins can act as the primary “bio-effectors” of metastasis [309], or to identify the epithelial, mesenchymal, and stemness biomarkers to characterize the CTC subpopulations [274]. Secretomics and mass spectrometry (MS)-based proteomics combinatorial analysis identified the stromal proteome of BC, including the protein–protein interaction (PPI) network, according to luminal-like and basal-like phenotypes, emphasizing secreted proteins that are increased by hypoxia [310]. The genomic landscape of cancer and the evolution during treatment may be non-invasively assessed by biomolecular characterization of CTCs [304]. Next generation sequencing (NGS)-based strategies allowed sequencing cancer genomes to reveal the subclonal diversification of primary BC [311]. The development of whole genome amplification (WGA) followed by NGS, microarray-based comparative genome hybridization (array-CGH), and single-circulating tumor cell sequencing techniques are able to profile single CTC [304]. Moreover, genomics-based approaches showed that plasma circulating cell-free DNA (cfDNA) and CTCs from the same blood sample provided complementary mutation information [312].

On the other hand, BC cells, primary tumor stroma and specific stromal components in distant organs intercommunicate and dictate the continuum of metastatic process through exosomes. These nano-vesicles are crucial mediators that transfer molecular signals/bioactive molecules, such as lipids, proteins, and different types of RNAs, such as microRNAs (miRNAs), mRNAs, transfer RNA, ribosomal RNA, small nuclear RNA, small nucleolar RNA, piwi-interacting RNA and long-non coding RNA, which are essential for intercellular communication [313]. Thus, tumor-derived exososmes contribute to generation of pre-metastatic niches, sustain the cancer dissemination, colonization, survival, proliferation or dormancy of incoming metastatic tumor cells, like in the case of BC cell-derived exosomes that transfer miR-21 to osteoclasts, promoting BC bone metastatic lesions [314]. Miller et al. (2021) sustained that even dormancy is a continuum of dormancy phenotypes characterized by hypometabolism, reduced feeding, reproduction or proliferation [213]. Thus, cancer cells exhibit a continuum of states from quiescence to long-term dormancy characterized by lack of cell division.

We previously reviewed and defined a Breast Cancer Cell Continuum Concept (BCCCC) comprised of successively integrated and interactive populations of heterogeneous tumor and CACs and sustained by a Breast Cancer Proteomics Continuum Concept (BCPCC), where each phenotype of neoplastic and CACs is characterized by a changing and adaptive proteomics profile [194]. Some authors delineated an epigenetic monoclonal progression continuum from normal to benign to invasive BC, based on DNA hypermethylation profiles [315]. Sanati et al. (2019) sustained that ductal carcinoma in situ (DCIS) has a continuum of histologically diverse proliferations that range from very well to very poorly differentiated [316]. Evidence emphasized the transition between an epithelial to a more mesenchymal cell state as an EMT continuum based on a continuum of multiple intermediate phenotypes of EMT transformation [317,318,319,320]. Thus, single-cell spatial transcriptomics based on RNA-sequencing has been preferentially used to characterize the EMT continuum [320,321].

**Table 2:** Vannote’s River Continuum Concept (RCC) applied to BC development (Breast Cancer Cell Continuum Concept (BCCCC) and Breast Cancer Proteomic Continuum Concept (BCPCC)).

RCC [293]				BCCCC and BCPCC [194]			
Longitudinal Changes in the Benthic Communities in Temperate Rivers				Longitudinal Changes in Kinetics of Metastasis			
ecological zonation	gradient of physical variables [322]	gradient of biological communities	gradient of energy input	BC progression	gradient of physical variables	BCCCC	BCPCC
headwaters or crenon	water temperature, flow, and oxygen level are low	shredders, collectors, grazers, predators	CPOM	primary breast tumor	PO_2_: 2.5–28 mm Hg (mv 10 mm Hg) [251]/<0.1–5% [252]; MR: 100,000–1,200,000 W/m^3^ [254]; temperature with 1.79 ± 0.88 °C higher that of the surrounding tissue [255]; pHi: 7.3–7.6 & pHe: 6.5–6.9 [258]	tumor cells; stromal cells (CAFs, TECs, TAPs, CAAs); immune cells (TAMs, TAMCs, TANs, TALs, TAPs, MDSCs); surrounding normal cells (luminal and myoepithelial cells); ECM	stem-like markers (CD44^high^/CD24^low^, EpCAM, PI3K, ALDH1^+^)
MIGRATION/DRIFT/COLONIZATION				EMT/INTRAVASATION			
rhithron	high water current and dissolved oxygen; low temperature	collectors, grazers, shredders, predators	FPOM, UPOM	bloodstream or lymphatic vessels	blood: oxygen level 12% [252]	CTCs	epithelial markers (EpCAM, E-cadherin, CKs, ZO, ESPR1); mesenchymal-like markers (N-cadherin, VIM, Twist1, AKT and PI3K, ZEB1); stemness-like markers (ALDH1, CD44, gangliosides, ABC proteins) [274]
MIGRATION/DRIFT/COLONIZATION				ETRAVASATION/MET/COLONIZATION			
potamon	low speed; low oxygen content; sandy river bed; higher water temperature; higher bacterial density	collectors, predators	FPOM, UPOM	preferred BC distant metastatic sites	bone: oxygen levels <1–6% (7–43 mmHg) [286]	homing and dormant DTCs	overexpression of epithelial markers (E-cadherin, occludin, crumbs3); downregulation of mesenchymal markers [323]

Abbreviations: ALDH1—aldehyde dehydrogenase-1; AKT—protein kinase B; BCCCC—Breast Cancer Cell Continuum Concept; BCPCC—Breast Cancer Proteomic Continuum Concept; CAAs—cancer-associated adipocytes; CAFs—cancer-associated fibroblasts; CKs—cytokeratins; CPOM—coarse particulate organic material; CTCs—circulating tumor cells; DTCs—disseminated tumor cells; ECM—extracellular matrix; EMT-epithelial-mesenchymal transition; EpCAM—epithelial cellular adhesion molecule; ESPR1-epithelial splicing regulator 1; FPOM—fine particulate organic material; pHe—extracellular pH; pHi—intracellular pH; MDSCs—myeloid-derived suppressor cells; MET—mesenchymal-epithelial transition; MR—metabolic rate; mv—median value; PI3K –phosphoinositide 3-kinase; PO_2_—oxygen partial pressure; RCC—River Continuum Concept; TALs—tumor-associated lymphocytes; TAMCs—tumor-associated mast cells; TAMs—tumor-associated macrophages; TANs—tumor-associated neutrophils; TAPs –tumor-associated pericytes; TECs—tumor endothelial cells; TICs—tumor-initiating cells; TME—tumor microenvironment; UPOM—ultra-fine particulate organic material; VIM—vimentin; ZEB1—zinc finger E-box binding homeobox 1; ZO—zonula occludens.

## 4. Conclusions

From a clinical-based point of view, breast cancer (BC) is the most common neoplasm in women, characterized by the invasion and metastasis hallmarks as the most defining features of BC malignancy [324]. From a more complex biomedical perspective, BC is characterized as a disease of the genome, epigenetic disease, environmental disease or ecological disorder, as well as a problem of developmental biology. Therefore, many mechanisms of cancer progression have been explained by principles of ecology, developmental biology, and evolutionary paradigms. Many authors have discussed ecological, developmental, and evolutionary strategies for more successful anti-cancer therapies or for understanding the ecological, developmental, and evolutionary bases of BC exploitable vulnerabilities. In terms of the eco-evolutionary theories, BC cells can function as a reprogrammed unicellular “pseudo-organism” able to selfishly survive within high stressful environments with ancestral features, while the BC tumor can be hyperbolically viewed as a “pseudo-organ”, or living organism able to build a self-sustainable tumor ecosystem, population, species, local community, biocenosis or evolving dynamical ecosystem (i.e., immune or metabolic ecosystem) that emphasizes both developmental continuity and spatio-temporal change. Tumors have been also characterized as evolutionary islets, islands or archipelagos-like ecosystems, as well as integrate lake-catchments units, that undergo evolutionary and spatio-temporal dynamic processes that shape the tumor microenvironment (TME) and drive cancer cells migration. Moreover, a cancer cell community, also known as an oncobiota, has been described as a non-sexually reproducing species, as well as migratory, or invasive species, that expresses an intelligent behavior, or endangered and parasite species that fights to survive, to optimize its fitness inside the host’s ecosystem, to exploit or to disrupt its host’s circadian cycle, for improving the own proliferation and spreading. Breast tumors, as well as all living systems, have as general features, the complexity, hierarchical organization, self-renewal capacity, and ability to reprogram their phenotype, metabolism and behavior in order to adapt or to cope with environmental challenges. At the organismal level, BC initiation and progression are considered short-term microevolutionary processes, in which cells acquire transformative, proliferative and metastatic capabilities and are subject to selection promoted by surrounding tissue ecology or the restrictive pre-metastatic and metastatic niches in the body, such as the site of the primary tumor, circulatory system, and diverse organotropic metastatic sites, respectively. BC could be also characterized as an ecosystem of co-evolving clones, competing and cooperating with each other and with cancer-associated cells (CACs), the TME becoming an arena of competition, predation, parasitism, and mutualism. Furthermore, disseminated tumor cell (DTCs) populations could be eradicated using similar eco-evolutionary dynamics of Anthropocene species extinctions as an alternative model for cancer treatment. Recent studies have established branched evolution as a feature of cancer and compared the evolution of BC in the human body to the origin of new species from a common ancestor organism, according to Darwin’s theory. Thus, according to the ecological speciation theory, carcinogenesis can also be viewed as a form of speciation, because cancer cells share several characteristics with conventional species. Cancer is also considered a by-product of multicellularity, so many authors have emphasized that cancer progression is a complex eco-evolutionary process, suggesting that the understanding of cancer’s evolutionary history could effectively help to monitor, manage and treat cancer. Similarities between microbial and cancer cells, phylostratigraphy, (onco)-phylogenomics, and (onco)-paleogenomics converge towards the hypothesis of cancer cells that reactivate an ancestral genetic program that allows them to adopt a series of multicellular-to-unicellular life reversions. Thus, cancer cells reactivate those genes that confer them the ability to survive toxic and stressful environment, proliferate abnormally, become immortalized, de-differentiate, de-specialize, de-construct, invade, migrate, colonize other niches and, probably, to recapitulate early forms of life. Other evidence sustains the use of ecological vulnerabilities of TME to improve BC treatment, similar to starvation and climate change in nature that drive the species extinction. BC tumorigenesis has also been compared with both the early embryo and placenta development that may suggest new strategies for research and therapy.

In conclusion, eco-evolutionary theories are essential for understanding BC development. Herein, we used the integrated framework of three well known ecological theories: Bronfenbrenner’s theory of human development, Vannote’s River Continuum Concept (RCC), and the Ecological Evolutionary Developmental Biology (Eco-Evo-Devo) theory, to explain and understand several eco-evo-devo-based principles that govern BC genesis and progression. These integrated eco-evo-devo theories can help clinicians to better diagnose and treat BC, for example by using of non-invasive biomarkers in liquid-biopsies, emerged from integrated multi-omics-based data that accurately reflect the biomolecular landscape of the primary tumor, in order to avoid the mutilating preventive surgery, such as bilateral mastectomy. From the perspective of preventive, personalized, and participatory medicine, these hypotheses may help patients to think about this disease as a process governed by natural rules, to understand the possible causes of the disease, and to gain control of their own health.

## Data Availability

Not applicable.
